# Aberrant SKP1 Expression: Diverse Mechanisms Impacting Genome and Chromosome Stability

**DOI:** 10.3389/fcell.2022.859582

**Published:** 2022-03-08

**Authors:** Laura L. Thompson, Kailee A. Rutherford, Chloe C. Lepage, Kirk J. McManus

**Affiliations:** ^1^ CancerCare Manitoba Research Institute, CancerCare Manitoba, Winnipeg, MB, Canada; ^2^ Department of Biochemistry and Medical Genetics, University of Manitoba, Winnipeg, MB, Canada

**Keywords:** cancer, centrosome dynamics, chromosome instability, DNA damage response, Fbox protein, genome instability, SCF complex, SKP1

## Abstract

The S-phase Kinase-Associated Protein 1 (SKP1) is a core component of the SKP1, Cullin 1, F-box protein (SCF) complex, an E3 ubiquitin ligase that serves to poly-ubiquitinate a vast array of protein targets as a signal for their proteasomal degradation, thereby playing a critical role in the regulation of downstream biological processes. Many of the proteins regulated by SKP1 and the SCF complex normally function within pathways that are essential for maintaining genome stability, including DNA damage repair, apoptotic signaling, and centrosome dynamics. Accordingly, aberrant SKP1 and SCF complex expression and function is expected to disrupt these essential pathways, which may have pathological implications in diseases like cancer. In this review, we summarize the central role SKP1 plays in regulating essential cellular processes; we describe functional models in which *SKP1* expression is altered and the corresponding impacts on genome stability; and we discuss the prevalence of *SKP1* somatic copy number alterations, mutations, and altered protein expression across different cancer types, to identify a potential link between SKP1 and SCF complex dysfunction to chromosome/genome instability and cancer pathogenesis. Ultimately, understanding the role of SKP1 in driving chromosome instability will expand upon our rudimentary understanding of the key events required for genome/chromosome stability that may aid in our understanding of cancer pathogenesis, which will be critical for future studies to establish whether SKP1 may be useful as prognostic indicator or as a therapeutic target.

## Introduction

The SKP1 (S-phase Kinase-Associated Protein 1), CUL1 (Cullin 1), F-box protein complex (SCF complex) is an E3 ubiquitin ligase that regulates a vast array of cellular processes (e.g., cell cycle, DNA damage response, apoptosis and centrosome homeostasis) that are key to maintaining genome stability and ensuring proper segregation of genetic material into daughter cells. SKP1 is an invariable, core component of the SCF complex that functions as the adaptor protein responsible for binding CUL1 and recruiting various F-box proteins for SCF complex formation. This critical role of SKP1 enables the poly-ubiquitination of a diverse array of substrates targeted by the variable F-box proteins for subsequent proteolytic degradation by the 26S proteasome, making SKP1 activity essential to regulate the myriad of cellular processes governed by the SCF complex. Accordingly, genetic aberrations altering SKP1 expression and/or function will adversely impact the many biological processes normally required to maintain genome stability, and thus aberrant *SKP1* expression is predicted to contribute to cancer pathogenesis. In support of this possibility, somatic alterations in *SKP1*, including mutations, deletions and mRNA misexpression occur frequently in a wide variety of cancer types.

Despite the many associations between altered *SKP1* expression and cancer, the fundamental impact aberrant SKP1 expression and/or function has on oncogenesis remains unclear. This review describes how aberrant SKP1 expression and function impacts many biological pathways that are essential to maintain genome instability that when altered, are implicated in oncogenesis. Accordingly, these observations support the possibility that aberrant *SKP1* expression may be a contributing pathogenic event, although definitive empirical data are still needed. First, we provide a historical background of mammalian SKP1, describing key characteristics at the gene/protein level as well as its relationship with orthologs from other species. We then discuss how SKP1 interacts with the other SCF complex members and their collective role within the ubiquitin proteasome system (UPS). Next, we describe the roles that SKP1 and the SCF complex have within three biological processes that are essential for maintaining genome stability, an enabling hallmark of cancer (Hanahan and Weinberg, 2011) including: 1) altered DNA damage response and apoptosis; 2) aberrant centrosome duplication and dynamics; and 3) chromosome stability. To further support a potential role in cancer pathogenesis, we detail the occurrence and frequency of *SKP1* alterations within cancer patient samples. Finally, we conclude with a brief discussion on future therapeutic strategies that seek to exploit altered *SKP1* expression and the downstream impacts of aberrant protein targeting and destruction.

## SKP1—A Historical Perspective and Fundamental Properties

Mammalian SKP1, also referred to as the Cyclin-A/Cyclin Dependent Kinase (CDK) 2-Associated Protein 19 (P19), was originally identified in 1980 within the guinea pig organ of corti by 2D polyacrylamide gel electrophoresis and was consequently named Organ of Corti Protein 2 (OCP2) (Thalmann et al., 1980; Thalmann et al., 2003). In the 1990s, a series of research groups independently investigated *SKP1/P19* and its aliases *OCP2* and *TCEB1L* as seemingly distinct genes. In 1995, Zhang and others (Zhang et al., 1995) determined that human SKP1/P19 interacted with the Cyclin A/CDK2 complex, suggesting a potential role in cell cycle regulation, and subsequently sequenced the *SKP1/P19* DNA coding regions. Concurrently, Chen *et al* (Chen et al., 1995) sequenced human *OCP2*, while Sowden *et al* (Sowden et al., 1995) presented the cDNA sequence for a novel gene designated *TCEB1L*, suspected to encode a transcription elongation factor. Additionally, Bai and others (Bai et al., 1996) identified the yeast and human orthologs of SKP1 as a suppressor of cdc4 (cell division control 4) and as a Cyclin F-binding protein, respectively, in two independent lines of research. It was not until 1997, when Liang *et al* (Liang et al., 1997) noted that the coding sequences detailed above for human *SKP1/P19*, *OCP2*, and *TCEB1L* were identical and that the above genes encoding distinct roles in diverse cellular processes were in fact, one and the same.

**FIGURE 1:**
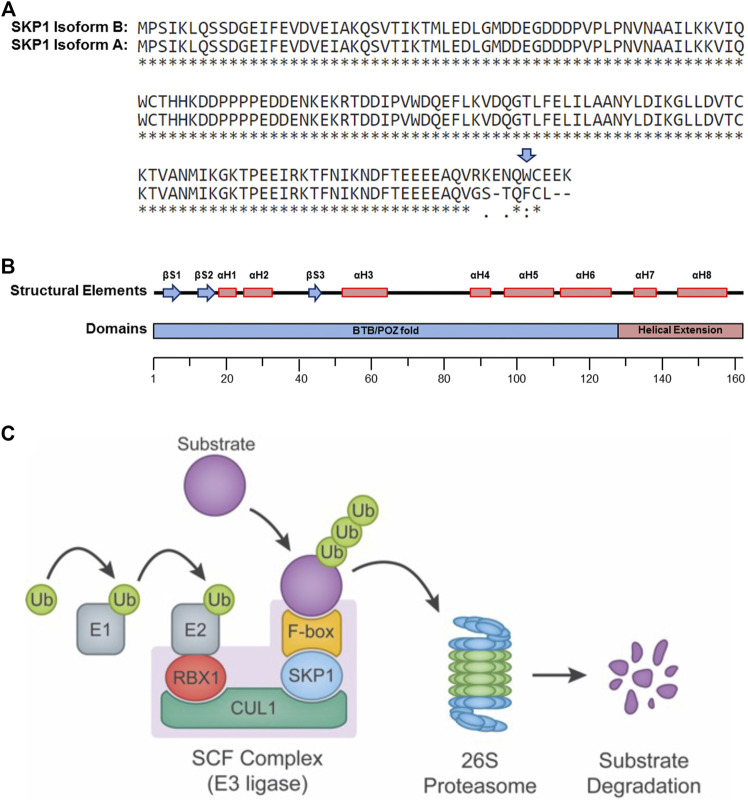
SKP1 Structure and Function. **(A)** Single amino acid sequence alignment of the two SKP1 (isoform A and isoform B) reveals sequence divergence within their carboxy-terminal tails. Sequence alignments performed using UniProt (Universal Protein Resource) (Altschul et al., 1990). Tryptophan 159 (W159), present only within Isoform B is highlighted by a blue arrow. A “*” identifies conserved amino acid positions, while “:” and “.” identify amino acid positions with similar or weakly similar properties, respectively. **(B)** Schematic depiction for the secondary structural elements (top) and protein domains (bottom) of SKP1 isoform B (βS, beta-sheet; αH, alpha-helix; BTB/POZ, broad complex, tramtrack and bric-à-brac(BTB)/poxviruses and zinc finger (POZ)). **(C)** Diagram depicting the SCF complex and its function in targeting protein substrates for poly-ubiquitination and proteolytic degradation by the 26S proteasome. The SCF complex consists of three invariable components (RBX1, CUL1, and SKP1) and one of 69 variable F-box proteins that confers substrate specificity. In general, ubiquitin (Ub) moieties are transferred to a protein substrate through the sequential actions of an E1 (activating) and an E2 (conjugating) enzymes in conjunction with an E3 (ligase) enzyme (e.g., SCF complex).

The human *SKP1* gene spans a region of 28,097 base pairs (bp) on chromosome 5q31.1 and encodes two protein coding mRNA transcripts of different lengths, 2,028 bp and 2,714 bp that are generated by alternative splicing. The transcripts are translated into two protein isoforms, 163 (Isoform B) and 160 (Isoform A) amino acids in size that differ at their carboxy-terminal regions (Figure 1A) (2009). Although Isoform B is considered the prototypic SKP1 protein (Schulman et al., 2000; Yamanaka et al., 2002; Kong et al., 2004), the potential functional differences between the two isoforms have yet to be fully explored. Nevertheless, a study in *Saccharomyces cerevisiae* revealed that the tryptophan residue at position 159 (Trp159), present only in human Isoform B (Figure 1A), is essential for its *in vivo* function. As Trp159 is evolutionarily conserved from yeast to humans, these experimental findings in *S. cerevisiae* suggest there may only be one functional human isoform (i.e., Isoform B) (Schulman et al., 2000). To test this possibility, isoform-specific studies must be designed to formally interrogate the functional differences and discern whether the non-prototypic SKP1 Isoform A has developed a *de novo*, Trp159-independent function during evolution.

SKP1 Isoform B (Figure 1B) is ∼18 kDa and harbors a 128 residue domain at the amino-terminus resembling the α-helix/β-sheet structure of a BTB/POZ (broad complex, tramtrack and bric-à-brac/poxviruses and zinc finger) fold domain, but with an α-helical insertion (αH4) (Schulman et al., 2000). This domain is essential for heterodimerization and is required for the binding of SKP1 to the SCF complex scaffolding protein, CUL1. Additionally, SKP1 harbors a two-helix, carboxy-terminal extension (αH7 and αH48) that cooperates with elements of the BTB/POZ fold to create a variable interaction motif that binds F-box domains (Figure 1B). There are 69 distinct proteins containing F-box domains (i.e., F-box proteins) that have been identified in mammals (Jin et al., 2004), each with its own set of protein targets. Thus, SKP1 serves as an adaptor between CUL1 and one of 69 F-box proteins, playing a critical role in the formation of up to 69 distinct SCF complexes (Figure 1C) (Ng et al., 1998; Yoshida et al., 2011) and the regulation of a diverse set of protein targets and pathways.

## Evolution of SKP1 Sequence and Function From Model Organisms to Humans

The amino acid sequences and structural elements of human SKP1 share a significant degree of amino acid sequence similarity with its counterparts in model organisms including *S. cerevisiae* (98% similar; 43% identical), *Mus musculus* (100% similar; 99% identical), *Drosophila melanogaster* (100% similar; 77% identical), *Caenorhabditis elegans* (97% similar; 71% identical) and *Arabidopsis thaliana* (71% similar; 58% identical) (2009). Beyond these sequence and structural similarities, functional conservation is also readily apparent between humans and model organisms. For example, human *SKP1* has been shown to functionally compensate for *Skp1* deletion in *S. cerevisiae* (Bai et al., 1996). Although only one functional isoform is proposed to exist in humans, studies in *C. elegans* have identified at least 21 *SKP1* paralogs or *Skp1*-related genes, each exhibiting varying degrees of sequence similarity with human *SKP1* (Yamanaka et al., 2002). Similarly, *D. melanogaster* and *A. thaliana* harbor 7 and 19 *Skp1-*related genes, respectively (Yamanaka et al., 2002; Kong et al., 2004), which exhibit tissue-specific expression and unique binding specificities for both F-box and Cullin-family proteins. Furthermore, while the role of the shorter human SKP1 Isoform A has not yet been well-characterized, it remains possible that Isoform A may recognize alternate F-box proteins or be involved in SCF complex-independent functions. In general, the high degree of sequence and functional conservation throughout evolution underscores the key role SKP1 plays within the SCF complex and further emphasizes the importance of SKP1 in the regulation of fundamental cellular processes.

## SKP1 is a Core Component of the SCF Ubiquitin Ligase Complex and the Ubiquitin Proteosome System

SKP1 and the SCF complex are arguably best understood for their roles in poly-ubiquitination, proteolytic degradation and the UPS. The UPS is a highly coordinated series of events involving the covalent attachment of ubiquitin molecules to protein targets and the subsequent degradation of these poly-ubiquitinated targets by the 26S proteasome. Substrate poly-ubiquitination is accomplished through the successive and repeated activities of three key enzymes (Figure 1C) that are generically referred to as the E1 ubiquitin (activating) enzyme, the E2 ubiquitin (conjugating) enzyme and the E3 ubiquitin (ligating) enzyme (reviewed in (Hershko and Ciechanover, 1998; Nakayama and Nakayama, 2006; Deshaies and Joazeiro, 2009)). Approximately 600–650 E3 ligases are predicted to exist within humans, which impart the extensive and requisite specificities to regulate the hundreds to thousands of protein targets believed to be modulated by the UPS, whereas only two E1 and approximately thirty E2 enzymes exist within the human genome (Deshaies and Joazeiro, 2009).

The E3 ubiquitin ligases are classically divided into three main families based on distinct structural motifs and include: 1) the Really Interesting New Gene (RING)-finger family containing ∼600 members in humans; 2) the Homologous to the E6-AP Carboxyl Terminus (HECT) family having ∼30 human members; and 3) the RING-between RING-RING (RBR) family with ∼12 members in humans (Morreale and Walden, 2016). The RING-finger family is further divided into sub-families, which includes the Cullin-RING ligase subfamily. The SCF complex is often considered the prototypic Cullin-based RING-finger E3 ubiquitin ligase and is comprised of three invariable core components (Figure 1C): 1) the RING-finger protein RBX1 (Ring-Box 1, also known as the regulator of cullins 1 [ROC1]) that recruits the E2 ubiquitin-conjugating enzyme; 2) CUL1, a scaffolding protein that complexes the E2 to the SCF complex; and 3) SKP1, the adaptor protein that physically connects the F-box protein and corresponding protein target with the core SCF complex.

F-box proteins are classified into three distinct families according to their substrate recognition domains, namely FBXW, FBXL, and FBXO family members, which harbor WD40 repeats (e.g., FBXW7), leucine-rich repeats (e.g., FBXL1/SKP2) or other domains (e.g., FBXO28), respectively, (Jin et al., 2004). As indicated above, it is the F-box protein that imparts the protein target specificity to the SCF complex, with F-box proteins often binding to phospho-activated targets. Once bound to the protein target, the F-box protein/protein target are subsequently recruited to the core SCF complex through an interaction with SKP1 to enable the transfer of ubiquitin from an E2 conjugating enzyme onto the protein target. It is the repeated covalent attachment of ubiquitin moieties (i.e., poly-ubiquitination) via specific linkages (lysine 48 [K48] linkages) that label the designated substrates for degradation by the 26S proteasome. Thus, it is the UPS that regulates the global and temporal abundance of an extensive array of protein targets within a given cell (Kulathu and Komander, 2012).

While there are potentially 69 distinct SCF complexes, the substrates and functions for many of these SCF complexes remain largely unknown. Nevertheless, there are a few well characterized F-box proteins/SCF complexes that target key proteins involved in a variety of cellular pathways such as DNA damage repair, apoptosis, centrosome biology and chromosome stability (discussed below), which highlights their innate roles in maintaining genome stability and preserving mitotic fidelity. As such, future studies aimed at functionally characterizing the complete cellular repertoire of SCF complexes will be essential to advance our rudimentary understanding of the specific impact each individual SCF complex has in normal cell physiology and genome stability. Perhaps even more important will be the fundamental and clinical studies aimed at determining the impact aberrant expression and function of SCF complex components have on disease development. Indeed, aberrant SKP1 expression and/or function is already associated with several human genetic disorders, including Sjögren’s syndrome (a chronic inflammatory autoimmune disease) (Sandhya and Danda, 2014), sporadic Parkinson’s disease (a neurological degenerative disorder) (Mandel et al., 2012) and cancer (Silverman et al., 2012). Thus, defining the underlying molecular etiology giving rise to SKP1 (and SCF complex) dysfunction will be critical to ultimately determine the individual and collective impacts on disease pathology, especially as it potentially relates to cancer development and progression.

## SKP1 and the SCF Complex Coordinate the DNA Damage Response and Apoptosis

The processes that regulate cell cycle progression and DNA damage response are intimately linked and are essential to maintain genome stability. In the presence of genotoxic stress or a stalled replication fork, a cell cycle arrest is invoked to facilitate repair prior to cell cycle re-entry with the ultimate goal of preventing genomic damage (mutations and alternations) from being propagated within daughter cells (Bassermann et al., 2014). These processes are highly dependent on appropriate protein turnover that is regulated by the UPS. Indeed, the SCF complex, and therefore SKP1, exhibit key roles within the DNA damage response, some of which are detailed below.

In general, following a DNA double strand break, a checkpoint kinase, either ATM (Ataxia Telangiectasia Mutated) or ATR (Ataxia Telangiectasia and Rad3 Related) is auto-phosphorylated, which initiates a series of cascading phosphorylation events on downstream targets. For example, ATM initiates a G1 arrest by phosphorylating Cyclin D1, which is subsequently ubiquitinated by SCF^FBXO4^ and targeted for proteolytic degradation. In turn, Cyclin D1 degradation promotes CDK2 inhibition by releasing P21 from CDK4 (Agami and Bernards, 2000), which ultimately prevents E2F transcription factor activation and cyclin expression (Silverman et al., 2012). Alternatively, an S-phase or G2 arrest can be invoked through ATR phospho-activation of CHEK1 (Checkpoint Kinase 1), which is mediated by the adaptor protein Claspin (Mamely et al., 2006) to hyperphosphorylate CDC25A, labeling it for SCF^βTrCP(FBXW11)^ mediated targeting and proteolytic degradation to attenuate CDK activation (Busino et al., 2003). This CDK attenuation induces a cell cycle arrest, providing the requisite time for efficient DNA repair. Moreover, to ensure an adequate supply of deoxyribonucleotides for DNA repair, degradation of RRM2 (Ribonucleotide Reductase Regulatory Subunit 2) via SCF^CyclinF(FBXO1)^ is inhibited by ATR-mediated Cyclin F degradation (D’Angiolella et al., 2012). Concurrently, the pre-replication complex component, CDT1 (Chromatin Licensing and DNA Replication Factor 1) is targeted for degradation by SCF^SKP2(FBXL1)^ to prevent replication of damaged DNA (Kondo et al., 2004), while protein translation is reduced by the phospho-inactivation of the elongation factor, eEF2 (Eukaryotic Translation Elongation Factor 2) by eEF2K to prevent unnecessary energy expenditure during the DNA damage response. Once DNA repair is complete, SCF^βTrCP^ directs eEF2K degradation to rapidly resume protein synthesis (Kruiswijk et al., 2012). SCF^βTrCP^ also coordinates cell cycle re-entry by targeting phosphorylated Claspin for degradation, preventing CHEK1 activation by ATR, allowing for CDC25A reactivation of CDKs, while the increased abundance of CHEK1 is reduced by targeted degradation mediated by SCF^FBXO6^ (Silverman et al., 2012; Bassermann et al., 2014).

As the SCF complexes described above are crucial for DNA damage repair and maintaining genome stability, it is not difficult to envision how mutation, aberrant expression and/or function of SKP1 promotes genome instability and may contribute to cancer development and progression. For example, the siRNA-based silencing of βTrCP in S-phase cells exposed to ionizing radiation results in CDC25A accumulation (Jin et al., 2003), a defective S-phase check-point, failure to inhibit DNA replication and the propagation of DNA damage underlying genome instability and cancer (Bassermann et al., 2014).

In the event of excessive DNA damage, apoptosis is typically initiated to remove those cells from the population and prevent transmission of damaged DNA to daughter cells, which is a process normally regulated by the SCF^FBXW7^ complex. In response to DNA damage, GSK3 (Glycogen Synthase Kinase 3) phosphorylates the anti-apoptotic BCL2 (B-Cell Chronic Lymphocytic Leukemia/Lymphoma 2) family member MCL1 (Myeloid Cell Leukemia 1), allowing for SCF^FBXW7^-mediated MCL1 poly-ubiquitination and degradation. The cell death promoters BAX (BCL2 Associated X Protein) and BAK (BCL2 Antagonist/Killer) are released from MCL1 inhibition, which stimulates mitochondrial membrane permeabilization, caspase activation and apoptosis induction. Deletion of *FBXW7* or its functional inactivation in acute lymphoblastic leukemia (ALL) cells, impairs MCL1 degradation in response to DNA-damaging agents, resulting in MCL1 overexpression and evasion of apoptosis (Inuzuka et al., 2011). In support of a role in oncogenesis, *FBXW7* is somatically altered in >30% of human T-cell lymphomas, while T-cell-specific *Fbxw7* knockout mice develop ALL (Crusio et al., 2010). Moreover, ∼20% of patients with colorectal adenocarcinoma have somatic *FBXW7* mutations (Tate et al., 2019), with altered *FBXW7* expression contributing to tumor development and progression, while loss-of-function mutations are predicted to be deleterious. Furthermore, ∼50% of somatic *FBXW7* mutations occur at three hotspot codons (Arg465; Arg479; Arg505), which disrupt binding of FBXW7 to target substrates (Akhoondi et al., 2007; Cancer Genome Atlas Network, 2012; Grim, 2014), highlighting the critical role of the SCF complex and how dysregulation of key components may contribute to oncogenesis. Collectively, the above data demonstrate that SKP1 and the SCF complex are critical for coordinating a cellular response to DNA damage and facilitating either DNA repair or apoptosis depending on the extent of the damage.

As SKP1 is an invariable component of each SCF complex described above, *SKP1* alterations such as mutations or copy number alterations (gains or losses) are predicted to impede DNA damage repair and foster cell survival by adversely impacting pro-apoptotic pathways leading to genome instability and perhaps promoting oncogenesis. This possibility is supported by the work of Piva and others (Piva et al., 2002), who generated and employed a transgenic mouse expressing a *Cul1* deletion mutant (Cul1-N252) that sequesters and inactivates murine Skp1 (discussed further below). Interestingly, the *in vivo* inhibition of Skp1 function in a T-cell lineage corresponded with the development of T-cell lymphomas. Upon closer scrutiny, the authors also noted significant increases in micronucleus formation (DNA containing, extranuclear bodies indicative of DNA damage and genome instability (Bhatia and Kumar, 2013)), centrosome abnormalities, aberrant chromosome segregation and karyotypic heterogeneity. These data suggest SKP1 is critical to preserve the function of essential biological processes (e.g., DNA repair and apoptosis), while aberrant SKP1 expression and/or function disrupts these essential processes in a manner that may promote oncogenesis. Thus, it will be of tremendous interest to determine whether the accumulation of DNA damage within *SKP1*-deficient cancer cells or appropriate mouse models are associated with increased sensitivity towards genotoxic agents or whether these cells/models can be selectively targeted with immune checkpoint inhibitors or precision-based therapeutic strategies.

## SKP1 and the SCF Complex Regulate Centrosome Dynamics

To ensure the accurate and faithful transmission of genetic material to daughter cells, chromosome dynamics are tightly regulated by the UPS, which coordinates centriole/centrosome duplication and separation. Centrosome aberrations lead to ongoing chromosome missegregation events and aneuploidy that are frequently observed in a myriad of cancer types. For example, one immunohistochemical study (Pihan et al., 1998) revealed that 93% (81/87 total) of human breast, prostate, lung, colon, brain, and metastatic cancer samples exhibit abnormal centrosome phenotypes including aberrant size, shape, and numbers relative to those in noncancerous adjacent tissues. Moreover, the aberrant phenotypes observed in tumor-derived cell lines are correlated with CIN (chromosome instability), a common form of genome instability characterized by ongoing changes in chromosome number and/or structure that is an established driver of cell-to-cell and genetic heterogeneity (reviewed in (Geigl et al., 2008; Lepage et al., 2019; Vishwakarma and McManus, 2020)). More recent studies have determined that SKP1 localizes to the centrosome throughout the cell cycle and that SCF^CyclinF^ (D'Angiolella et al., 2010), SCF^FBXW5^ (Puklowski et al., 2011) and SCF^βTRCP^ (Chan et al., 2008) exhibit key roles in centrosome dynamics (Gstaiger et al., 1999; D'Angiolella et al., 2010) that when disrupted with proteasome inhibitors (MG132), adversely impact centrosome formation and duplication. For example, during G2, the centriolar protein CCP110 (Centriolar Coiled-Coil Protein 110) that normally promotes centriole replication while inhibiting elongation, is targeted for proteolytic degradation by SCF^CyclinF^ (Chen et al., 2002). Such timely CCP110 degradation prevents centriole over-duplication that would otherwise result in supernumerary centrosomes, chromosome missegregation events and aneuploidy. Indeed, D’Angiolella and others (D'Angiolella et al., 2010) determined that Cyclin F silencing induces centrosome over-duplication in G2 leading to multi-polar spindle formation, lagging chromosomes and an increase in micronucleus formation, all of which are hallmarks of CIN (Geigl et al., 2008; Lepage et al., 2019; Vishwakarma and McManus, 2020). As expected, co-silencing Cyclin F and CCP110 rescues these aberrant phenotypes effectively confirming the underlying mechanism leading to their formation.

Beyond CCP110, the centriolar scaffolding protein SASS6 (Spindle Assembly Protein 6) is also essential for centrosome formation and duplication, and is degraded in G2 by SCF^FBXW5^, which prevents over-duplication of centrosomes. FBXW5 is negatively regulated by APC/C (Anaphase-Promoting Complex/Cyclosome) and PLK4 (Polo-Like Kinase 4), which enables SASS6 to function appropriately during G1 and S-phase, respectively. As predicted, reduced *FBXW5* expression corresponds with increasing SASS6 abundance and abnormally increased numbers of centrioles (Puklowski et al., 2011). Similarly, PLK4 promotes centriole duplication and separation, and is tightly regulated by SCF^βTRCP^ (Guderian et al., 2010). Thus, aberrant PLK4 expression is associated with aberrant centriole numbers in human cancer cells (Habedanck et al., 2005), while *βTrcp1* knockout in mouse embryonic fibroblasts corresponds with centrosome over-duplication and supernumerary centrosomes (Guardavaccaro et al., 2003). SCF^βTRCP^ also contributes to centrosome homeostasis and chromosome stability by regulating the degradation of BORA (BORA Aurora Kinase A Activator), an activator Aurora Kinase A (AURKA). BORA regulates AURKA localization and kinase activity at the centrosome to ensure proper centrosome and mitotic spindle development, as overexpression of a SCF^βTRCP^-resistant form of BORA interferes with bipolar spindle formation as it adversely impacts AURKA localization and function (Chan et al., 2008). Based on these few examples, it is apparent that SKP1 and the SCF complex are critical for regulating centrosome dynamics and function, which is essential for chromosome transmission fidelity. Thus, further clinical studies into the types and prevalence of genomic aberrations affecting SKP1 expression are essential to better understand their impact on centrosome biology and gain a more holistic understanding of the potential downstream implications for disease development.

## Aberrant *SKP1* Expression Induces CIN That May Promote Oncogenesis

As an invariable component of the SCF complex, it is apparent that SKP1 is essential for the proper regulation of key substrates involved in many cancer-associated pathways. Despite this association, the potential pathophysiological impact aberrant *SKP1* expression may have in cancer development is only beginning to emerge. This knowledge gap may in part, be attributed to the lack of transgenic or *Skp1* knockout mouse models available for *in vivo* study (Zhou et al., 2013). Nevertheless, several transgenic mouse models do exist for the other SCF complex components (e.g., Cul1) that have provided key insight into SKP1 (and SCF complex) function, which includes the pathogenic implications for genomic instability and cancer associated with aberrant SCF complex expression and function. As indicated above, Piva *et al.* (Piva et al., 2002) developed a *Cul1* deletion mutant (Cul1-N252) transgenic mouse model that inactivates Skp1 *in vivo*, leading to lymphoid organ hypoplasia, proliferation defects, supernumerary centrosomes, mitotic spindle aberrations and CIN. Following the initial proliferation reduction, >80% of Cul1-N252 mice develop T-cell lymphomas, suggesting Skp1 and SCF function are required to prevent lymphoid tumor development. Moreover, Cul1-N252 expression in a human cellular context (HEK293T cells) resulted in many aberrant phenotypes associated with CIN, including multinucleated cells, enlarged nuclei and increased micronucleus formation. Thus, their mouse and human work are consistent with aberrant Skp1/SKP1 function being an early etiological event underlying CIN and possibly contributing cancer pathogenesis. Moreover, these results highlight the utility of mouse models for studying the *in vivo* functions of SCF components and provide a means by which to investigate their potential roles in tumorigenesis. Their findings also underscore the paucity of clinically-relevant *Skp1* mouse models, which are essential to clearly delineate and characterize any potential role for aberrant *Skp1*/*SKP1* expression and/or function in oncogenesis.

Recently, several genetic studies have begun to identify potential pathogenic relationships between aberrant SCF complex expression/function and cancer (Thompson et al., 2020; Bungsy et al., 2021; Lepage et al., 2021). In particular, two studies focused on the impact reduced *SKP1* expression has on CIN in colorectal (Thompson et al., 2020) and ovarian (Lepage et al., 2021) cancer contexts. First, Thompson *et al* (Thompson et al., 2020) performed a screen of 164 candidate genes whose diminished expression was suspected to underlie CIN. Using siRNA-based silencing and quantitative imaging microscopy, they determined that reduced *SKP1* expression induced significant increases in CIN-associated phenotypes (Lepage et al., 2019), such as nuclear areas, micronucleus formation and chromosome numbers. They further showed that *SKP1* silencing corresponded with increases in replication stress, DNA double strand breaks and chromothriptic events, or extensive chromosome shattering followed by reassembly in a single event (reviewed in (Ly and Cleveland, 2017)). Perhaps most importantly, they performed genetic rescue experiments and determined that the aberrant phenotypes were largely dependent on aberrant increases in Cyclin E1 levels, an established target of the SCF complex; however, as complete phenotypic rescues did not occur, they posited that additional protein targets must also be misregulated that contribute to the plethora of aberrant phenotypes observed. Given that ∼85% of sporadic colorectal cancers exhibit CIN (Lengauer et al., 1997; Cisyk et al., 2015; Cisyk et al., 2018), these findings are particularly important as they may shed new insight into the potential underlying molecular etiology driving colorectal cancer pathogenesis. A second study by Lepage and others (Lepage et al., 2021), assessed the impact that reduced *SKP1* (and *CUL1*) expression has on CIN in non-transformed fallopian tube secretory epithelial cells, a cell of origin for high-grade serous ovarian cancer (Perets et al., 2013; Nakamura et al., 2018). Using a combination of siRNA and CRISPR/Cas9 approaches, they demonstrated that reduced expression corresponded with significant changes in nuclear areas, micronucleus formation and chromosome numbers. They further showed that CIN was prevalent and dynamic over an ∼3-month timeframe, which is key given recent evidence showing that CIN is both pervasive and dynamic in ascites (an accumulation of abdominal fluid containing tumor cells) and solid tumor samples isolated from patients with high-grade serous ovarian cancer (Penner-Goeke et al., 2017; Morden et al., 2021). Collectively, these data identify *SKP1* as a novel CIN gene and further suggest that reduced expression may contribute to cancer pathogenesis. Accordingly, future fundamental and clinical studies are now essential to determine the extent and types of *SKP1* genetic alterations that may drive disease development and progression, with potential downstream implications for treatment response and patient outcomes.

## *SKP1* Expression is Frequently Altered in Human Cancers

As SKP1 and the SCF complex normally function to regulate a multitude of essential cellular pathways required to maintain genome stability, genetic alterations impacting the invariable complex components (e.g., *SKP1*) are anticipated to promote cellular dysfunction, which may contribute to cancer development. As detailed above, several genetic studies performed in both malignant (Thompson et al., 2020) and non-malignant (Lepage et al., 2021) human cell contexts have established that reduced *SKP1* expression induces CIN, an enabling hallmark of cancer (Hanahan and Weinberg, 2011) associated with cellular transformation, intra-tumoral heterogeneity, metastasis, drug resistance and poor patient outcomes (reviewed in (Geigl et al., 2008; Vishwakarma and McManus, 2020)). Unfortunately, *Skp1* knockout mice do not exist, suggesting it may be an essential gene, a possibility supported by a CRISPR screen that identified *SKP1* as an essential gene (Blomen et al., 2015); however, it should be noted that this work was conducted in a haploid malignant cancer cell line, and thus, the results may exhibit context-specific essentiality. Nevertheless, additional evidence comes from DepMap (Dependency Mapping), which is an online resource that identified *SKP1* a common essential gene based on RNAi and CRISPR screens performed in a myriad of cell lines (Tsherniak et al., 2017; Dempster et al., 2019; Dharia et al., 2021; Pacini et al., 2021). Accordingly, while *SKP1* appears to be an essential gene the functional impacts altered *SKP1* expression has on various biological pathways are only beginning to emerge (Thompson et al., 2020; Lepage et al., 2021).

**FIGURE 2:**
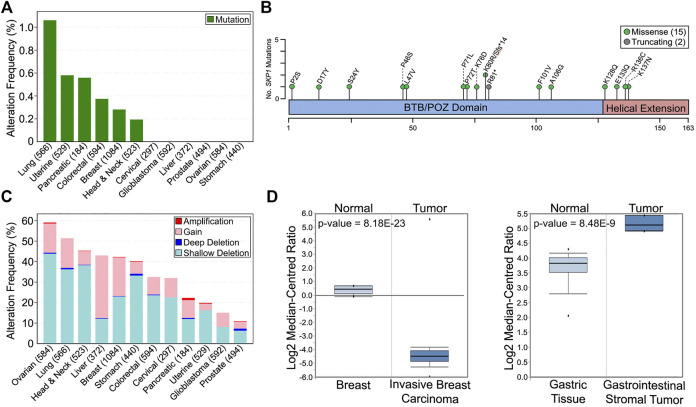
Prevalence and Impact of *SKP1* Alterations in Cancer. **(A)** The frequency of total *SKP1* mutations (missense; truncating; inframe; fusion) in 12 common, solid tumor cancer types (total cases) (Cerami et al., 2012; Gao et al., 2013). Note that only missense (15) and truncating (2) mutations were identified from the 12 pan-cancer TCGA datasets (Hoadley et al., 2018). **(B)** Schematic mapping the positions of the encoded *SKP1* mutations across the SKP1 protein using the corresponding single amino acid codes (fs, frameshift; *, premature stop codon). **(C)** Prevalence of *SKP1* copy number alterations (deep deletion; shallow deletion; gain; amplification) within the 12 common cancer types (total cases) (Cerami et al., 2012; Gao et al., 2013; Hoadley et al., 2018). **(D)** Box-and-whisker plots displaying *SKP1* mRNA expression levels for normal and tumor tissues from invasive breast carcinoma (left) and gastrointestinal stromal tumor (right). Boxes display interquartile range, whiskers denote 10th and 90th percentiles, and the minimum/maximum values are displayed as black dots. Note that a significant >25-fold decrease in mean *SKP1* expression occurs in invasive breast carcinoma relative to normal tissue, while a significant ∼3-fold increase in expression occurs in gastrointestinal stromal tumors. Data, graphs and statistical analyses were obtained from the Oncomine database (https://www.oncomine.org) (Rhodes et al., 2007).

In support of reduced *SKP1* expression and/or function harboring a potential pathogenic role in oncogenesis, *in silico* analyses of The Cancer Genome Atlas (TCGA) pan-cancer atlas patient data available through cBioPortal (Cerami et al., 2012; Gao et al., 2013) reveal that *SKP1* is somatically altered in 12 common solid tumor cancer types (Figure 2) (Hoadley et al., 2018). Briefly, *SKP1* mutations are rare with only 15 missense and 2 truncating mutations (one frameshift and one premature stop codon) identified within six of the 12 cancers assessed (Figure 2A) (Hoadley et al., 2018). Interestingly, and in agreement with *SKP1* being a putative tumor suppressor gene, the mutational load is equally distributed (i.e., diffuse) across the entire coding sequence (Figure 2B), rather than a focal mutational load that is typical of an oncogene (Liu et al., 2011; Vogelstein et al., 2013; Sato et al., 2015). With respect to gene copy number alterations, both gains (oncogene-like) and losses (tumor suppressor-like) occur in all 12 cancer types; however, losses are more prevalent in 11 of 12 cancers evaluated (Figure 2C). Overall, *SKP1* amplifications (two or more additional copies) are rare (0–1.0%), while gains (one additional copy) occur in all 12 cancers analyzed and range from 3.2 to 30.7% in uterine and liver cancers, respectively. Similarly, deep (i.e., homozygous) deletions are rare (0–1%), whereas shallow (i.e., heterozygous) deletions are present in all 12 cancer types and range from 6.3 to 43.8% in prostate and ovarian cancers, respectively. Collectively, these data show that large copy number alterations (amplifications or deep deletions) are rare, which suggests an expression threshold may exist whereby too much expression (i.e., gene amplification) may severely impact normal cellular physiology. Furthermore, complete loss (i.e., deep deletion) appears incompatible with viability further supporting the notion that *SKP1* is an essential gene (Blomen et al., 2015; Tsherniak et al., 2017; Dempster et al., 2019; Dharia et al., 2021; Pacini et al., 2021).

**FIGURE 3:**
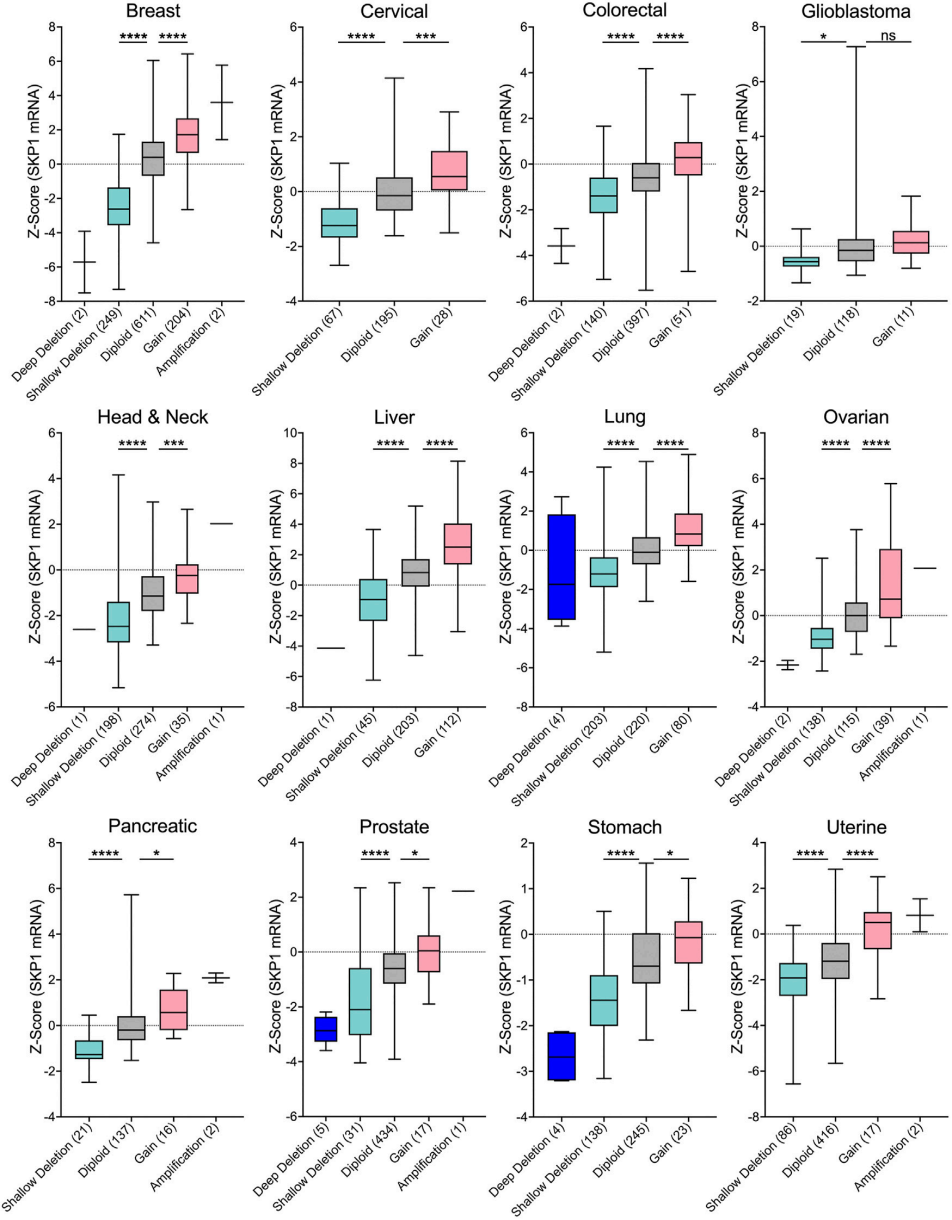
*SKP1* Copy Number Alterations are Positively Correlated with mRNA Expression Levels in Cancer. Box-and-whisker plots of TCGA pan-cancer data from 12 common cancer types reveal linear correlations between *SKP1* copy number alterations and mRNA expression levels (Cerami et al., 2012; Gao et al., 2013; Hoadley et al., 2018). Boxes identify interquartile ranges (25th, 50th, and 75th percentiles), while whiskers depict entire range. For orientation purposes, the dotted horizontal lines identify 0. Specific copy number alterations (deep deletion; shallow deletion; gain; amp) and diploid categories are presented along the *x*-axis with the total number of samples indicated within brackets. Unpaired t-tests were conducted comparing either Shallow Deletions or Gains with the corresponding Diploid control (ns [not significant] *p*-value >0.05; * *p*-value <0.05; *** *p*-value <0.001; **** *p*-value <0.0001). Note that in general, very few deep deletions or amplifications were identified in the 12 cancer types.

A fundamental assumption of gene copy number alterations is that they induce corresponding changes in gene expression and that *SKP1* copy number gains and losses are expected to underlie aberrant SCF complex activity leading to cellular dysfunction, genome instability and potentially tumorigenesis. Indeed, strong positive correlations exist between copy number changes and mRNA expression for all 12 cancer types investigated (Figure 3), and while the copy number alterations detailed above suggest *SKP1* may encode both oncogene-like or tumor suppressor-like functions, these seemingly opposing activities are not specific to *SKP1* and have been reported for other genes including *TP53* (Lane, 1984; Jenkins et al., 1985; Finlay et al., 1989), *USP22* (Jeusset and McManus, 2017), and *RAD54B* (McAndrew and McManus, 2017).

The potential for *SKP1* to encode both tumor suppressor-like and oncogene-like activities is further bolstered by the many additional gene expression datasets available through various online resources. For example, while data contained within the *In Silico* Transcriptomics Online database (https://ist.medisapiens.com) (Kilpinen et al., 2008) show tremendous variation in *SKP1* (ENSG00000113558) mRNA expression in both normal and tumor tissues (see (Thompson et al., 2021)), they also reveal that some cancers exhibit increases (head and neck; chronic lymphocytic leukemia; liver) or decreases (breast; ovarian; cervical; colorectal) in *SKP1* expression relative to the corresponding normal tissues. Additionally, expression data from the Oncomine database (https://www.oncomine.org) (Rhodes et al., 2007) corroborate that *SKP1* can be under or overexpressed within specific cancer types relative to normal tissues. For example, Figure 2D provides representative examples in which *SKP1* is predicted to encode both tumor suppressor-like functions, as mRNA expression is significantly reduced (∼25-fold) within invasive breast carcinomas relative to normal tissues, or oncogene-like functions, as expression is significantly increased (∼3-fold) within gastrointestinal stromal tumors. Collectively, the data presented above support the possibility that *SKP1* may encode either oncogene- or tumor suppressor gene-like capabilities depending on whether it is over or under-expressed, respectively.

Unfortunately, very little insight into *SKP1* expression is available beyond transcriptomics, as only a single study has been performed in which SKP1 was assessed at the protein level. In 2015, Liu and others (Liu et al., 2015) employed western blots (64 matched cases) and immunohistochemistry (20 matched cases) to investigate SKP1 expression in non-small cell lung cancer and adjacent normal lung tissues. While both approaches revealed variable SKP1 expression in both cancer and matched tissues, 56% of cases showed significant increases in expression within tumors relative to control tissues. Furthermore, they determined that SKP1 expression was inversely correlated with survival as patients with high expression levels had significantly worse overall survival than those with low expression levels; however, the thresholds defining high versus low were not specified. Although the underlying genomic defects accounting for the increases in SKP1 expression observed in this study were not determined, this single example supports the possibility that aberrant SKP1 expression may be a pathogenic driver of cancer.

Collectively, the above data gleaned from a diverse array of patient-based genomic, transcriptomic and protein datasets show that *SKP1* is frequently misexpressed in human cancers, which suggests aberrant SKP1 expression may harbor tumor suppressive or oncogenic functions depending on whether it is under- or over-expressed, respectively. These apparently opposing activities may simply reflect that as a core SCF complex member, SKP1 may function as a tumor suppressor or oncoprotein depending on the protein targeted for degradation suggesting SKP1 expression levels may need to be precisely regulated to maintain cellular homeostasis, preserve genome stability and prevent cancer development and progression. Thus, the patient-based findings presented above underscore the need for additional insight into SKP1, its protein targets and the underlying biological mechanisms and their potential impact for oncogenesis. In this regard, future studies should also assess the clinical utility of *SKP1* as a potential prognostic indicator or a novel therapeutic target for cancers.

## SKP1 and the SCF Complex as Potential Therapeutic Targets in Cancer

As the SCF complex regulates a diverse array of substrates involved in many biological pathways fundamental to genome stability, therapeutically targeting a core SCF component such as SKP1 may seem counter intuitive as there is the potential for increased toxicity and side effects. However, therapeutic success has been achieved with general proteasome inhibitors (e.g., Bortezomib (Robak et al., 2015)) and indirect SCF inhibitors (e.g., MLN4924 (Swords et al., 2015)) for the treatment of lymphoma, myeloma and leukemia lending support to use of broad-spectrum inhibitors targeting SKP1 and/or the SCF complex (Skaar et al., 2014). In fact, evidence shows cancer cells with a misregulated UPS are more sensitive to the broad-spectrum proteasome/SCF-targeting inhibitors than non-cancerous cells, which allows for the use of lower drug concentrations for effective outcomes and reduced side effects (Ludwig et al., 2005). Based on these findings, SKP1-targeted therapies designed to block SCF complex formation and function may represent promising treatment options. Rather than inhibiting global proteasomal degradation with agents like Bortezomib, or inactivating additional off-target Cullin family members with MLN4924, SKP1 inhibitors would specifically target the SCF complex, thereby reducing toxicity and ideally enhancing the therapeutic window (Silverman et al., 2012). Although a clinically administered dose would need to be strictly monitored, SKP1/SCF complex inhibitors could potentially be utilized in combination regimens with other chemotherapies to improve efficacy and/or help reduce the risk of drug resistance. For example, 5-fluorouracil, oxaliplatin, and irinotecan are first-line chemotherapies that induce DNA damage and cellular apoptosis (Longley et al., 2003). These drugs are often administered in combination for the treatment of colorectal cancer, with response rates from 40–50% and improved median survival (Douillard et al., 2000; Giacchetti et al., 2000; Longley et al., 2003). As the SCF complex is critical for eliciting an effective DNA damage response, perhaps co-treatment with a low-dose SKP1/SCF complex inhibitor would further sensitize cancer cells and synergize with standard chemotherapies to improve response rates and patient outcomes.

Considering the frequency of *SKP1* copy number losses in cancer (Figure 2C), it remains plausible that a synthetic lethal (SL) paradigm may prove highly effective in a broad range of cancer types. Synthetic lethality is defined as a rare and lethal genetic interaction occurring between two unlinked genes. In practice, cells harboring a mutation in either gene alone remain viable, whereas the presence of both mutations within a single cell will induce lethality (Sajesh et al., 2013). Although a relatively new therapeutic concept, SL strategies have already begun to enter the clinic as breast and ovarian cancers harboring *BRCA1/2* (Breast Cancer Type 1/2 Susceptibility Protein) defects are now being targeted with PARP1 (Poly [ADP-Ribose] Polymerase 1) inhibitors like Olaparib. Accordingly, genetic studies aimed at identifying SL interactors of *SKP1* are highly warranted as the SL interactors are candidate drug targets that when inhibited are predicted to induce the selective killing of cancer cells harboring *SKP1* defects. Beyond the genetic sensitization approaches detailed above, another promising strategy involves proteolysis-targeting chimeric molecules, or Protacs (reviewed in (Sakamoto et al., 2001; Burslem and Crews, 2020; Cecchini et al., 2021; Hughes et al., 2021)). The fundamental concept behind Protacs is that fusion proteins are created to link a specified target substrate to an F-box protein for SCF-mediated ubiquitination and degradation (Sakamoto et al., 2001). This approach would allow for conditional or tissue-specific degradation of overexpressed oncoproteins, suppression of tumor growth and cancer cell death.
